# Half Burr-Hole Method: A Novel Surgical Technique for Reducing Brain Shift and Improving Electrode Placement Accuracy in Deep-Brain Stimulation

**DOI:** 10.1055/a-2707-0593

**Published:** 2025-10-03

**Authors:** Yosuke Ito, Masafumi Fukuda, Tomoyoshi Ota, Hiroshi Masuda, Makoto Oishi

**Affiliations:** 1 Department of Neurosurgery, Nishi Niigata Chuo National Hospital, Niigata, Japan; 2 Department of Neurosurgery, Niigata University Brain Research Institute Clinical Neuroscience Branch, Niigata, Japan

**Keywords:** anterior thalamic nucleus, deep-brain stimulation, pneumocephalus

## Abstract

**Background:**

Deep-brain stimulation (DBS) is used to treat movement disorders and drug-resistant focal epilepsy. However, electrode placement accuracy is affected by brain shift caused by pneumocephalus and cerebrospinal fluid (CSF) leakage during surgery. We present the novel half burr-hole method for improved DBS electrode placement accuracy.

**Case Description:**

This approach was used to treat a 28-year-old man with drug-resistant epilepsy in whom stereo-electroencephalography revealed bilateral seizure onset in the temporal lobes, precluding focal resection. The patient, under general anesthesia, was placed in the supine position. Using a ROSA robot-assisted surgical system, approximately 8-mm-deep “partial burr-holes” were created, with the deeper portion perforated using a 2.4-mm twist drill. Stimulation electrodes were placed bilaterally in the anterior thalamic nucleus. Directional leads were secured using standard burr-hole caps. Postoperative computed tomography confirmed a 0.46-cm ^3^ pneumocephalus and electrode positioning with 0.47 mm (range: 0–1.62 mm) vector and 0.12 mm (range: 0.08–0.16 mm) axial errors relative to the target coordinates. Postoperative electrode impedance values were within the normal range.

**Conclusion:**

The half burr-hole method effectively minimizes CSF leakage and pneumocephalus during DBS surgery, reducing brain shift and enhancing electrode placement accuracy, and is compatible with standard burr-hole caps for electrode fixation, minimally affecting impedance values.

## Introduction

Deep-brain stimulation (DBS) is a standard treatment modality for movement disorders such as Parkinson's disease, tremor, dystonia, and drug-resistant epilepsy. 1 2 3 The success of DBS surgery depends on precise electrode placement at the tentative target, which is predetermined based on preoperative anatomical imaging. However, intraoperative brain shift—that is, brain tissue displacement owing to pneumocephalus and cerebrospinal fluid (CSF) loss during surgery—is a well-recognized cause of reduced targeting accuracy. 4 5 While various approaches have been suggested, 6 7 no definitive solution to this problem has been established in clinical practice.

Herein, we introduce the half burr-hole technique, a novel method developed to minimize brain shift during DBS targeting the anterior nucleus of the thalamus (ANT). In this technique, the burr-hole is drilled to half the normal skull thickness, followed by a twist-drill perforation and dural opening using monopolar cautery, which helps minimize CSF leakage. Furthermore, the Stimloc system is compatible with this technique, providing reliable postoperative electrode fixation.

## Case Presentation

### Case Illustration

The patient was a 28-year-old Japanese man with drug-resistant epilepsy who initially presented with focal impaired awareness seizures (FIAS) 20 years ago. The patient's clinical history is summarized in Table 1 . Neurological examination revealed no abnormalities, and the patient's cognitive function was intact. There were no comorbid conditions, and the patient had no history of previous surgeries. Antiepileptic medication was initiated 12 years after onset, resulting in good seizure control after 1 year. However, FIAS recurred 5 years ago, prompting a referral to our hospital the following year. Weekly seizures persisted despite medication adjustments, prompting bilateral temporal lobe stereo-electroencephalography evaluation 6 months ago. Focal resection was deemed unfeasible based on the intracranial recordings, which indicated that seizure onset originated from the lateral aspects of both temporal lobes. Consequently, ANT-DBS was selected as the treatment approach. Since ANT-DBS electrodes are implanted via a transventricular approach, there is a high risk of brain shift due to CSF leakage, which may compromise the accuracy of electrode placement. Therefore, this technique was selected.

**Table 1:** Clinical course to date

Age (y)	Clinical course
8	Onset of focal impaired awareness seizures (FIAS)
20	Initiation of antiepileptic drug (AED) therapy (12 y after onset)
21	Seizure control achieved after 1 y of treatment
23	Recurrence of FIAS
24	Referred to our hospital
28	Weekly seizures persisted despite multiple medication adjustments
27.5	Underwent bilateral temporal lobe stereo-electroencephalography
–	Seizures identified as originating from both lateral temporal lobes; resection unfeasible
28	ANT-DBS selected as the treatment approach

Abbreviations: ANT, anterior nucleus of the thalamus; DBS, deep-brain stimulation.

This study was approved by the Ethics Committee of Nishi-Niigata Chuo National Hospital. Written informed consent to participate in the study was obtained from the participant.

### Technique

**Fig. 1:**
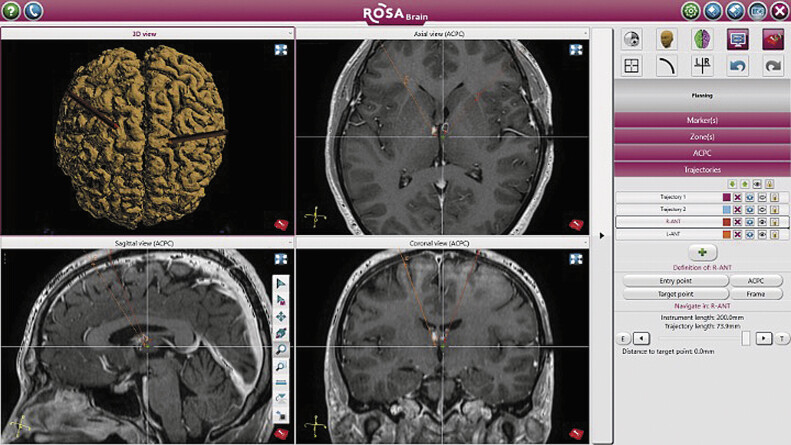
Coordinates of the tentative target (anterior nucleus of the thalamus, ANT) and insertion trajectory were determined using the ROSA planning software (Zimmer Biomet; Warsaw, Indiana, United States).

Preoperative determination of the tentative target (the ANT) and insertion trajectory was performed using the ROSA planning software (Zimmer Biomet, Warsaw, Indiana, United States; Fig. 1 ).

On the day of surgery, a Leksell frame was mounted on the patient's head using four pins, followed by cranial imaging using a computed tomography (CT) suite. In the operating room, the patient was positioned flat, and the cranial CT data were registered into the ROSA ONE robotic system (Zimmer Biomet).

**Fig. 2:**
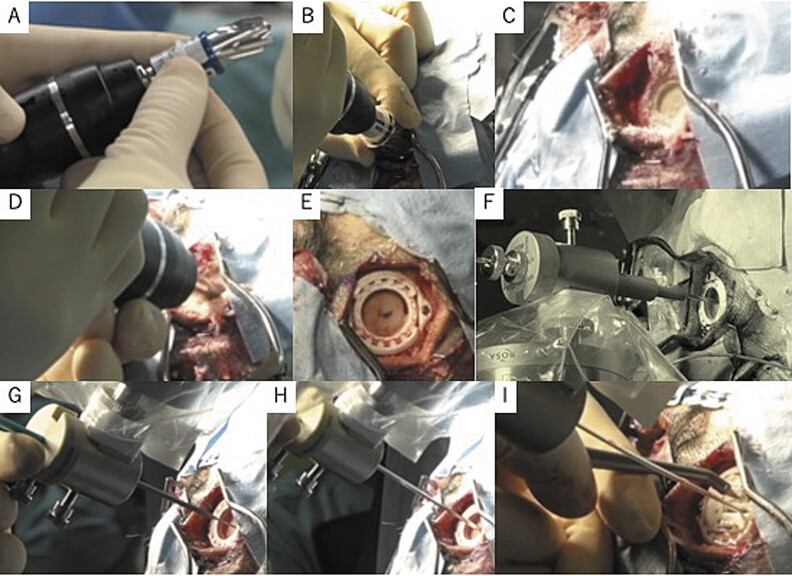
Surgical field image demonstrating the half burr-hole technique. ( **A, B** ). The initial partial burr-hole was drilled to a depth of approximately 8 mm, with the perforation depth controlled using a rubber ring attached to the drill adapter and a perforator guard (S & Brain Corporation, Tokyo, Japan) positioned on the cranial surface, serving as stoppers. ( **C, D** ). The resulting hole had a stepped floor: it was contoured flat using a specialized trephine (Rescue Round Cutter: S & Brain Corporation). ( **E** ) Stimloc bases were mounted onto bilateral half burr-holes. ( **F** ) The deeper cranial bone was perforated using a 2.4-mm twist drill. ( **G** ) The dura mater was penetrated and coagulated using an electrocautery device. ( **H** ) The insertion cannula was introduced. ( **I** ) The deep-brain stimulation (DBS) electrode was inserted and secured with the Stimloc.

Using the robotic arm of the system, the electrode insertion sites were marked bilaterally in the frontal region, 4-cm skin incisions were made, and the insertion points were re-marked on the exposed bone surface. Initial partial burr-holes were made to a planned depth of approximately 8 mm, with penetration controlled using a rubber ring attached to the drill adapter as a stopper, in tandem with a perforator guard (PG01: S & Brain Corporation, Tokyo, Japan; Fig. 2A ). Subsequently, a specialized trephine (Rescue Round Cutter, SPD103: S & Brain Corporation) was used to contour the stepped floor of the initial holes, enabling the attachment of the Stimloc device (Medtronic, Minneapolis, Minnesota, United States; Fig. 2B ).

Next, the ROSA guidance system was used again to mark the electrode insertion point. The skull was perforated using a 2.4-mm twist drill ( Fig. 2C ), and the dura mater and cortical surface were cauterized using a specialized electrosurgical scalpel. A catheter insertion needle (outer diameter: 2.1 mm; Elekta AB, Stockholm, Sweden) was advanced to the target point, and a biological tissue adhesive (BOLHEAL Tissue Sealant, KM Biologics, Kumamoto, Japan) was sprayed at the cortical entry point to prevent CSF leakage. Frontal and lateral radiographs were obtained to confirm that the position of the needle tip deviated < 1 mm from the tentative target. Subsequently, the inner catheter was removed, and a directional lead with integrated stylet was inserted (B33005M; Medtronic). The outer catheter was withdrawn, biological adhesive was reapplied, and the lead was secured using the Stimloc device ( Fig. 2D ). The appropriate placement of the electrodes was confirmed through a second series of intraoperative radiographs.

Following bilateral electrode placement, electrode impedance measurements and deep-brain recordings were performed. Magnetic resonance imaging was performed 6 days after the ANT-DBS surgery. Postoperative 3D-T1 images were imported into the ROSA planning software, and the deviation between the actual position of the electrode tip and its preoperatively planned position was measured. Postoperative CT examination revealed a pneumocephalus volume of 0.46 cm ^3^ , indicating minimal CSF leakage. The electrodes were accurately placed near the planned target coordinates.

**Fig. 3:**
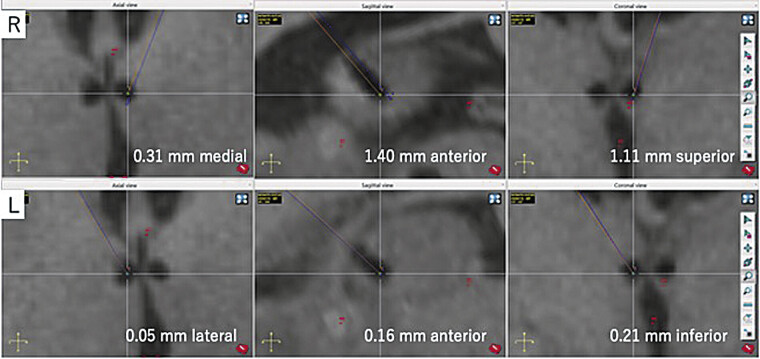
Postoperative electrode location deviations from the preoperative ROSA plan. Postoperative T1-weighted MRI shows electrode tip locations in axial, sagittal, and coronal views. Top row (R): right electrode position with deviations of 0.31 mm medial, 1.40 mm anterior, and 1.11 mm superior to the planned coordinates. Bottom row (L): left electrode position with deviations of 0.05 mm lateral, 0.16 mm anterior, and 0.21 mm inferior to the planned coordinates. The blue line represents the trajectory planned during preoperative ROSA planning. The orange line corresponds to the actual electrode trajectory, reconstructed based on the center of the hypointense region representing the electrode tip.

Deviation measurements based on postoperative MRI showed that the right electrode deviated by 0.31 mm medial, 1.40 mm anterior, and 1.11 mm superior, while the left electrode deviated by 0.05 mm lateral, 0.16 mm anterior, and 0.21 mm inferior to the preoperative plan ( Fig. 3 ). The total time from skin incision to the creation of bilateral half burr-holes was 32 minutes and from bilateral electrode placement to skin closure was 45 minutes. The patient was discharged on postoperative day 8. Stimulation was initiated on postoperative day 3 at 2(−)C(+) with 1.5 mA, 90 µs, and 145 Hz bilaterally. At the 3-month follow-up, the stimulation intensity was increased to 3.5 mA. No stimulation-related adverse effects, such as psychiatric symptoms or memory impairment, were observed. The surgical wound showed no abnormalities. Prior to surgery, the patient experienced FIAS 20 to 30 times per week. Following ANT-DBS, the seizure frequency decreased to approximately twice per week.

## Discussion

The half burr-hole technique proposed in this study is designed to suppress brain shift by minimizing CSF leakage using a small-diameter cranial and dural perforation. In addition, by preserving the lower half of the skull at the burr-hole site, this method eliminates the need for extradural hemostasis and sealing procedures typically employed to prevent CSF leakage. Furthermore, lead fixation using the Stimloc system is feasible with this approach, offering greater postoperative stability compared to titanium microplates, thereby reducing the risk of electrode displacement.

DBS is commonly performed via the burr-hole technique, in which a cranial burr-hole is created coaxially to the planned electrode trajectory. After dural incision and visualization of the cortical surface, the electrode is inserted under stereotactic guidance. However, intracranial pneumocephalus occurring during DBS surgery can lead to brain shift, thereby impairing the precision of electrode placement. 8 Several factors have been implicated in brain shift, including CSF leakage, intraoperative changes in intracranial pressure, and air influx into the cranial cavity. 4

To address these concerns, multiple technical refinements have been introduced. These include three-layer sealing of the arachnoid, pia, and cortex with fibrin glue, 8 burr-hole closure using polyethylene glycol-based sealants such as DuraSeal, 7 asleep DBS under general anesthesia, 9 11 and twist-drill craniostomy with a reduced diameter of 5 mm. 12 These approaches have significantly decreased pneumocephalus volume from 8 to 44 mL using conventional methods to as little as 1.3 to 12 mL. 13 More recently, Zhang et al introduced a pneumocephalus-minimizing technique (PMT) in which the dural opening is minimized and the burr-hole is sealed dynamically during insertion using Gelfoam, resulting in a drastic reduction of pneumocephalus surface area from 5.91 to 0.21 cm ^2^ . 11 We performed the procedure under general anesthesia with the patient's head maintained in a flat position, limiting postoperative pneumocephalus to a minimal volume of 0.46 cm ^3^ .

We adopted a modified half burr-hole technique in which the lower portion of the skull bone is preserved, thereby eliminating the need for postoperative burr-hole sealing. This approach also simplifies extradural hemostasis and allows for accurate targeting, even in cases with thick cranial bone or eccentric lead trajectories. Unlike conventional methods requiring burr-hole enlargement in such situations, 10 14 our method reduces the need for re-drilling by utilizing stereotactic twist drilling.

Electrode fixation is achieved using the Stimloc anchoring system, which offers superior stability compared to titanium microplate fixation, where greater postoperative displacement has been reported. 15 Thus, this technique ensures secure lead stabilization and minimizes the risk of migration.

As in Zhang's PMT, 11 direct visualization of cortical vasculature during lead insertion is not feasible. However, the safety of this approach is supported by data indicating that selecting an entry site more than 4 mm away from surface vasculature avoids hemorrhagic complications. Our method utilizes a conservative 5-mm safety margin, which we believe provides comparable safety. Table 2 summarizes the technical differences between the conventional burr-hole, PMT, twist drill, and the present half burr-hole technique.

**Table 2:** Comparison between the conventional method and the present technique

Item	Burr-hole technique	PMT technique	Twist drill Technique	Half burr-hole technique (this study)
Burr-hole diameter	∼14 mm	∼14 mm	∼3–5 mm	14 mm
Bone preservation	Full removal	Full removal	Perforation only	Lower half preserved
Dural penetration	Present	Minimally performed	Minimally performed	Minimally performed
CSF leak prevention	DuraSeal or PEG-based sealant	Dynamic sealing with Gelfoam	None	Fibrin glue
Pneumocephalus volume (ref.)	8–44 mL	0.21 cm ^2^	Relatively low	0.46 cm ^3^
Lead fixation	Titanium/plastic plate	Titanium/plastic plate	Plate or none	plastic plate
MER compatibility	◎ (multi-track possible)	△ (single-track only)	△ (single-track only)	△ (single-track only)
Extradural hemostasis required	Yes	Yes	No	No
Cortical vessel safety margin	Direct visualization	4 mm	Unclear	5 mm

We set the twist-drill perforation depth at 8 mm to ensure compatibility with all lead clips currently approved in Japan. While the superficial diameter achieved by the perforator reaches 14 mm, the deep diameter is limited to 11 mm, which may interfere with the anchoring mechanism of some lead clips. To address this, a rescue cutter is used to expand the deep-bone cavity; alternatively, a steel bar may serve as a substitute when necessary.

This technique is suitable for cases in which the cranial bone is ≥ 10 mm thick and multi-track MER is not essential.

### Limitation

This case highlights the potential utility of the half burr-hole technique in ANT-DBS; however, as a single case report, the findings should be interpreted with caution. Further large-scale studies should validate its reproducibility, safety, and long-term efficacy. If minor trajectory adjustments are required intraoperatively, they can be addressed either by additional twist-drill perforation or by creating a full burr-hole to the dura (PMT method).

## Conclusion

The half burr-hole technique proposed in this study effectively suppresses CSF leakage and minimizes brain shift. By preserving the lower skull margin at the burr-hole site, this approach eliminates the need for extradural hemostasis and sealing procedures. Furthermore, this technique allows for lead fixation using the Stimloc system, reducing the risk of postoperative electrode displacement compared to titanium microplates.
